# Self-efficacy as a mediator between health technology readiness and health-related quality of life: A survey study with mediation analysis

**DOI:** 10.1177/20552076261462636

**Published:** 2026-06-19

**Authors:** Anna Vahteristo, Virpi Jylhä, Hanna Kuusisto

**Affiliations:** 1 Department of Social and Health Management, 163043 University of Eastern Finland, Kuopio, Finland; 2 Department of Neurology, 60670 Tampere University Hospital, Tampere, Finland

**Keywords:** digital health, health technology readiness, digital health literacy, health-related quality of life, self-efficacy

## Abstract

**Background:**

Digital health supports self-management of preference-sensitive conditions and improves health-related quality of life (HR-QoL). Additionally, digital health literacy (DHL) is positively associated with HR-QoL, although not always directly, with self-efficacy as a mediator. Health technology readiness, as a broader concept, encompasses DHL and may further enhance understanding of these relationships.

**Objective:**

This study aimed to examine the relationship between health technology readiness and HR-QoL and the potential mediating role of self-efficacy in this association among people with preference-sensitive neurological conditions.

**Methods:**

The cross-sectional study used Finnish versions of the Readiness and Enablement Index for Health Technology (READHY-FIN), Self-Efficacy for Managing Chronic Disease (SEMCD-FIN), and EuroQol five-dimensional questionnaire (EQ-5D-5L) to assess health technology readiness, self-efficacy, and HR-QoL. A linear regression model was used to investigate associations between the constructs, and the PROCESS Macro for SPSS was used to test self-efficacy as a possible mediator.

**Results:**

The domains of health technology readiness: DHL (*B*=.156, p<.001), self-management (*B*=.296, p<.001), and social support (*B*=.118, p<.001), were positively associated with HR-QoL. However, the associations were indirect between DHL and HR-QoL (*B*=.163; p<.001), and between social support and HR-QoL (*B*=.122; p<.001), consistent with mediation through self-efficacy. Self-management had significant direct (*B*=.105, p=.003) and indirect (*B*=.191, p< .001) associations, consistent with partial mediation.

**Conclusions:**

Self-efficacy mediates the associations of DHL and social support with HR-QoL, whereas self-management had both direct and indirect associations. These findings emphasize a holistic approach of health technology readiness in the development of digital health.

## Introduction

Digital health is widely utilized in the management of chronic diseases.^1–3^ It enables participation in the self-care process and supports shared decision-making,^4^ in which patients actively engage in treatment decisions.^5^ This is essential in the care of preference-sensitive conditions, where multiple reasonable treatment options exist.^6,7^ Chronic neurological conditions like epilepsy and multiple sclerosis (MS) are preference-sensitive conditions,^8^ requiring individually planned regular monitoring and treatment.^9,10^ The growing number of digital health applications supporting the care of neurological diseases^11^ indicates that both patients and healthcare professionals are actively using them in care and self-management.^12^ These applications include digital care pathways and self-management applications to support the care of people with epilepsy^12,13^ and MS.^14–18^

Digital health literacy (DHL), the ability to search, evaluate, and use health-related information in digital form to handle or solve a health problem,^19^ has been shown to be associated with higher patient engagement with digital health services (DHS) for the self-management of chronic diseases.^20^ Although evidence is scarce, there is some indication that the active use of digital health applications is positively associated with perceived health and quality of life in the treatment of asthma,^21^ underactive thyroid,^22^ and multimorbidity.^23^ In addition, research shows that DHL is positively related to HR-QoL,^24–31^ a patient-reported outcome commonly used to assess patients’ perceived health.^32^ However, the mechanism underlying this relationship may involve explanatory factors such as health-promoting behaviors and self-efficacy as mediators.^30,33^ The role of perceived self-efficacy is to describe one’s confidence in their ability to change or manage their health-related habits.^34^ These and other self-reported outcome measures have been increasingly used in the evaluation of digital health.^35^

**Figure 1.**
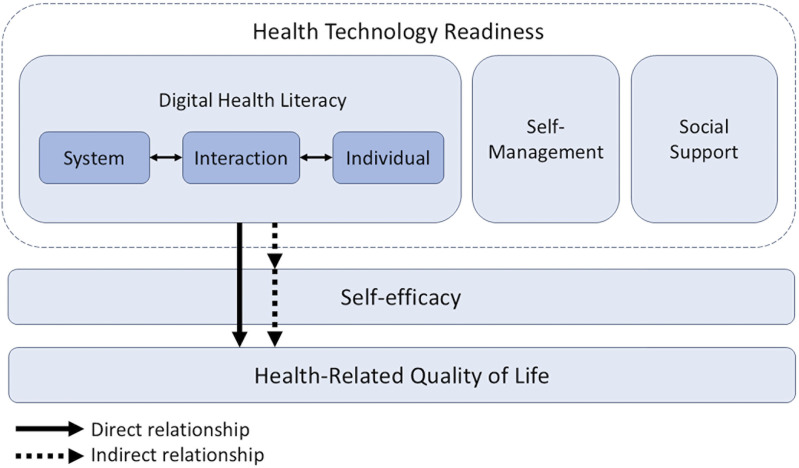
Relationships between health technology readiness, self-efficacy, and health-related quality of life based on the literature.

Recent research describes DHL as a comprehensive construct, comprising three interrelated aspects: the individual, the system, and their interaction.^36^ In addition, it is part of a composite construct of health technology readiness, alongside individuals’ self-management skills, and the experience of social support individuals feel they receive for their health (see Figure 1). Including self-management skills in the construct of health technology readiness provides further insight into individuals’ capabilities to manage their condition and emotional distress, as well as their disease burden. Moreover, social support is defined as the extent to which individuals feel supported by healthcare professionals, family, and peers; another factor affecting individuals’ ability to interact with health technology.^37^ Assessment of health technology readiness has been useful in identifying the readiness of individuals to use health technology among older adults^38,39^ and people with chronic conditions, e.g. type-2 diabetes,^40^ inflammatory bowel disease,^41^ chronic skin condition,^42^ and recipients of an implantable cardioverter defibrillator.^43^

In addition to the relationships among DHL, self-efficacy, and HR-QoL, there are indications that self-management and social support, also aspects of health technology readiness, are related to self-efficacy and HR-QoL. In general, social determinants, such as higher education and income, younger age, and social support, are positively associated with HR-QoL in populations with chronic diseases.^44^ Furthermore, in addition to the knowledge needed for decision-making about their healthcare, social support from family, peers, and healthcare providers is essential for self-management of chronic conditions.^45^ Moreover, interventions promoting social support have been reported to improve HR-QoL among people with MS.^46^ In addition, self-management skills are positively associated with self-efficacy among people with type 2 diabetes^47^ and hemodialysis.^48^ A positive association between self-management skills and HR-QoL was also identified, although with low statistical significance.^47,48^

With the increasing use of DHS to support the care and self-management of chronic conditions, it is essential to enhance the understanding of the factors positively associated with HR-QoL. In addition to understanding the positive relationship between DHL and HR-QoL and health technology readiness as a comprehensive construct of the ability to use digital health, the purpose of this research is to investigate the relationship between health technology readiness and HR-QoL among people with preference-sensitive neurological conditions. Therefore, in this study, we assessed (1) the relationship between health technology readiness and HR-QoL, and (2) the role of self-efficacy as a possible mediator between health technology readiness and HR-QoL among people with preference-sensitive neurological conditions. Figure 1 presents the relationships between DHL, self-efficacy, and HR-QoL, based on the literature presented above, and the construct of health technology readiness, under investigation.

## Methods

### Recruitment

A cross-sectional survey was conducted between April 2022 and April 2023 to investigate the relationships between health technology readiness, self-efficacy, and HR-QoL among people with preference-sensitive neurological conditions. People with epilepsy or multiple sclerosis (MS), were chosen, as they are typical neurological preference-sensitive conditions. Both are highly prevalent diseases affecting individuals of all ages. Moreover, there are DHS, e.g., patient interfaces, digital care pathways, and patient interfaces of neuro registries available for people with epilepsy or MS to support the care of their chronic condition. The survey was targeted at people diagnosed with epilepsy or MS. The diagnosis was used as the inclusion criterion, and no exclusion criteria were applied.

The first round of data collection took place from April to August 2022, as the Finnish Neuro Society shared an invitation to the online survey with its members. The second round included an invitation to the online survey, distributed by the Finnish Epilepsy Association to its members in November 2022 and by the Finnish Pensioners’ Federation in January 2023, aiming to reach its members diagnosed with epilepsy. These surveys were closed in March 2023. In addition, paper questionnaires were handed out at the events organized by the patient organizations and at the Department of Neurology in a university hospital in Finland. The paper questionnaire included a link to the electronic version of the survey, enabling respondents to complete it electronically if they chose to do so. The questionnaires were available in Finnish and Swedish, the official languages of Finland. 18 responses were received on a paper questionnaire. As there were no differences based on the response method, all responses are combined and analyzed together. In this study, sample size calculation was not feasible due to the recruitment strategy. The survey invitation was disseminated through patient organizations via social media and newsletters. Therefore, the total number of individuals with epilepsy or MS who had access to the invitation could not be determined. Consequently, it was not possible to conduct an a priori sample size calculation. Instead, we aimed to reach as many eligible participants as possible through these channels.

### Ethical considerations

The research was approved by the Ethics Committee of the Tampere University Hospital (R21057). The respondents were informed that their participation was voluntary, that anonymity was guaranteed, and that they could opt out at any time. Written informed consent was obtained from the participants after reading the information sheet and before entering the questionnaire. The research was conducted in accordance with the Finnish National Medical Research Act,^49^ the guidelines governing non-medical research,^50^ and the principles of good research practice.^51,52^ Following the ethical principles,^50^ it was possible to respond on behalf of a person when they were unable to do so themselves.

### Measures

The Finnish and Swedish versions of the Readiness and Enablement Index for Health Technology (READHY) instrument were used to assess health technology readiness. READHY instrument consists of 65 items that form five READHY domains describing health technology readiness from three different perspectives: DHL (individual, interaction, and system), self-management, and the social support individuals feel they receive from their healthcare providers, relatives, and peers. Each domain includes two to four dimensions, and the score for each dimension and domain can be calculated as the mean of the items in that domain. The items are rated on a four-point Likert scale (where 1 equals strong disagreement, and 4 equals strong agreement), and the item on emotional distress was reverse-scored so that a higher score indicates lower emotional distress.^37^ READHY-FIN has been translated, culturally tested, and validated with the Finnish population.^53^ READHY-SWE has been translated by the developers of the original READHY instrument.^37^

HR-QoL was assessed using the EuroQoL five-dimensional questionnaire (EQ-5D-5L),^54^ a reliable instrument used in different populations and settings.^55^ It includes five aspects of health status: mobility, self-care, usual activities, pain/discomfort, and anxiety/depression,^56^ and each of the five aspects can be reported separately, or by EQ-5D Index, a weighted value converted from individual EQ-5D profiles by using validated value sets suitable for the selected population. EQ-5D Index ranges between one (full health) and zero (condition equaling death). It cannot exceed 1 but can be less than 0 in health states considered worse than dead.^57^ As there is no country-specific EQ-5D Index value set for the Finnish population, we used the Danish value set by Jensen et al.^58^ as it is closest to the Finnish population socio-economically and culturally and has been used in previous Finnish research.^59–62^

In addition, we utilized the 6-item Self-Efficacy for Managing Chronic Disease scale (SEMCD),^63^ which we translated into Finnish, SEMCD-FIN, as part of this research. For the Swedish questionnaire, we used the SEMCD-SWE.^64^ The six items cover domains common in many chronic diseases, including symptom control, role function, emotional functioning, and communication with the physician. Each item has a scale ranging from 1 (less self-efficacy) to 10 (higher self-efficacy), and the score is the mean of the six items.^63^

The questionnaire was complemented with general background variables, including age, gender, and educational level. Furthermore, the respondents were asked about how long ago they were diagnosed with their neurological chronic condition, and about their use of DHS in the management of their chronic condition, including patient interfaces or digital care pathways for MS or epilepsy. Nonusers reported having never used DHS to manage their chronic condition.

### Statistical analysis

The data analysis was performed with SPSS (version 29.0; IBM Corp). The data was described with percentages and means. Pearson’s correlation coefficient was used to examine the relationships between READHY domains, self-efficacy, and HR-QoL. Analysis showed that the three domains describing the DHL aspect of the READHY tool (individual, interaction, and system) were strongly interrelated with correlations exceeding 0.7. Therefore, these items were combined into a summary variable of DHL, which was used for further analysis. Although in the literature the domains of DHL are reported separately to provide a comprehensive understanding of DHL, together they provide a profile of the respondent.^37^

Multivariate linear regression analysis was used to investigate associations among respondent characteristics, health technology readiness, self-efficacy, and HR-QoL. The pre-analytic process, including assessments of normality, linearity, and homoscedasticity,^65^ was performed prior to the analysis. Furthermore, collinearity diagnostics^65^ were performed before accepting variables into the final models. In addition, PROCESS Macro for SPSS (version 4.3), developed by Hayes,^66,67^ was used to test the mediating relationships of self-efficacy. It is applicable in similar research assessing mediating effects.^21,33^ The theoretical model includes two hypotheses: (1) health technology readiness has a positive relationship with HR-QoL, and (2) this relationship is mediated by self-efficacy. Path c´ refers to the direct association between health technology readiness on HR-QoL, and the product of *a* and b (a*b) to the indirect association between health technology readiness to HR-QoL through self-efficacy. In addition, Path *c* refers to the total association between health technology readiness on HR-QoL (c´+ a*b). Analysis was run separately for each of the three aspects of health technology readiness (DHL, self-management, and social support). Bootstrap estimation (10000 samples) implemented in the PROCESS macro was used to estimate the possible indirect effect.^66^ A significance level of .05 was used to assess the statistical significance of the results.

## Results

### Respondent characteristics

The total number of participants was 289, and their characteristics are presented in Table 1. Of the participants, 78.9% were women (228/289), and 39.4% (114/289) were aged between 40 and 54 years. Over half of the respondents (150/289, 51.9%) were diagnosed with epilepsy or MS over 10 years ago, and nearly two-thirds (188/289, 65.1%) were using digital health services in the care of their disorder.

**Table 1.** Respondent characteristics (N=289).

Sociodemographic variables	Values, n (%)
**Gender, n (%)**	
Woman	228 (78.9)
Man	59 (20.4)
Missing	2 (0.7)
**Age groups (years), n (%)**	
<30	20 (6.9)
30–39	47 (16.3)
40–54	114 (39.4)
55–64	66 (22.8)
>64	37 (12.8)
Missing	5 (1.7)
**Time from diagnosis of epilepsy or MS, n (%)**	
Less than 1 year ago	8 (2.8)
1–5 years ago	90 (31.1)
6–10 years ago	39 (13.5)
Over 10 years ago	150 (51.9)
Missing	2 (0.7)
**Educational level, n (%)**	
Primary and lower secondary education	22 (7.6)
Upper secondary or vocational education	88 (30.4)
Bachelor’s level degree	89 (30.8)
Master’s level or doctoral degree	87 (30.1)
Missing	3 (1.0)
**Use of digital health services for the care of disorder, n (%)**	
Active users (monthly or more often)	58 (20.1)
Occasional users (once or a few times a year)	130 (45.0)
Nonusers	100 (34.6)
Missing	1 (0.3)

Table 2 presents a descriptive summary of the key variables, including the READHY-FIN domains describing health technology readiness, the SEMCD-FIN for self-efficacy, and the EQ-5D Index for HR-QoL. The means of the five domains of health technology readiness ranged between 2.67 (social support) and 2.98 (DHL, Individual).

**Table 2.** Mean and SD of health technology readiness, self-efficacy, and health-related quality of life.

Variables	Mean	SD^a^
Health technology readiness		
Digital health literacy, Individual	2.98	0.54
Digital health literacy, Interaction (between individual and system)	2.86	0.53
Digital health literacy, System	2.76	0.59
Self-management	2.80	0.25
Social support	2.67	0.68
Self-efficacy	6.41	2.09
Health-related quality of Life	0.74	0.27

^a^standard deviation.

Univariate analysis indicates significant differences in health technology readiness among people with epilepsy or MS based on their educational level. Hence, those with higher educational levels also had higher health technology readiness. Similarly, we found significant differences in self-efficacy, with respondents having a higher educational level exhibiting higher self-efficacy. In addition, there was a significant difference in HR-QoL among respondent groups based on how long ago they were diagnosed with their disorder. However, in pairwise comparisons, this difference was significant only between those diagnosed 6 to 10 years ago compared to those diagnosed over 10 years ago.

### Association between health technology readiness and HR-QoL, and the role of self-efficacy

Correlations between health technology readiness domains, self-efficacy, and HR-QoL are presented in Table 3. All correlations were positive and significant. Although the correlation between self-management and self-efficacy was very high (*r=*.707, *P* < .001), pre-analysis and collinearity diagnostics supported including both variables in the linear regression model as separate predictors.

**Table 3.** Correlations between the domains of health technology readiness, self-efficacy and health related quality of life.

​	1	2	3	4	5
1. Digital Health Literacy	1	​	​	​	​
2. Self-management	*r*=.549	1	​	​	​
	*P*<.001				
3. Social support	r*=*.466	r=.560	1	​	​
	*P*<.001	*P*<.001			
4. Self-efficacy	*r*=.470	*r*=.707	*r*=.477	1	​
	*P*<.001	*P*<.001	*P*<.001		
5. Health-related Quality of Life	r=294	*r*=.543	*r*=295	*r=*.626	1
	*P*<.001	*P*<.001	*P*<.001	*P*<.001	

*r* = Pearson’s correlation coefficient.

*P*<.05 is considered statistically significant.

The results of the multivariate linear regression analysis are reported in Table 4. Model 1 confirms that the respondent characteristics did not significantly explain the variance in HR-QoL. In Model 2, which included health technology readiness, only *self-management* was significantly associated with HR-QoL (*B*=.325, SE=.038, *P* < .001). Model 2 explained 32.9% of the variance in HR-QoL. Adding self-efficacy to the model (see Model 3) increased the model’s explanation to 44.2%, as both self-management (*B*=.161, SE=.042, *P* <.001) and self-efficacy had significant positive associations with HR-QoL (*B*=.063, SE=.009, *P* <.001).

**Table 4.** Multivariate linear regression analysis of associations among health technology readiness, self-efficacy, and health-related quality of life.

Variables	Model 1 (health-related quality of life)			Model 2 (health-related quality of life)			Model 3 (health-related quality of life)		
	*B* ^a^	SE^b^	*P value* ^c^	*B* ^a^	SE^b^	*P value* ^c^	*B* ^a^	SE^b^	*P value* ^c^
Gender	.014	.040	.73	.017	.036	.63	.009	.032	.77
Age	-.001	.001	.51	-.001	.001	.19	-.001	.001	.40
Educational level	-.031	.018	.38	-.042	.015	.007	-.038	.014	.008
Time since diagnosis	.030	.017	.75	-.009	.016	.58	-.013	.015	.36
Digital health literacy	​			-.012	.036	.73	-.032	.033	.33
Self-management	​			.325	.038	<.001	.161	.042	<.001
Social support	​			-.004	-026	.88	-.023	.024	.34
Self-efficacy	​			​	​	​	.063	.009	<.001
*R*^2^ (*R*^2^ change)^d^	.022			.329 (+.307)			.442 (+.114)		
*P value* ^c^	.23			<.001			<.001		

^a^Unstandardized coefficient.

^b^Standard error.

^c^*P*<.05 is considered statistically significant.

^d^Adjusted R Square.

**Figure 2.**
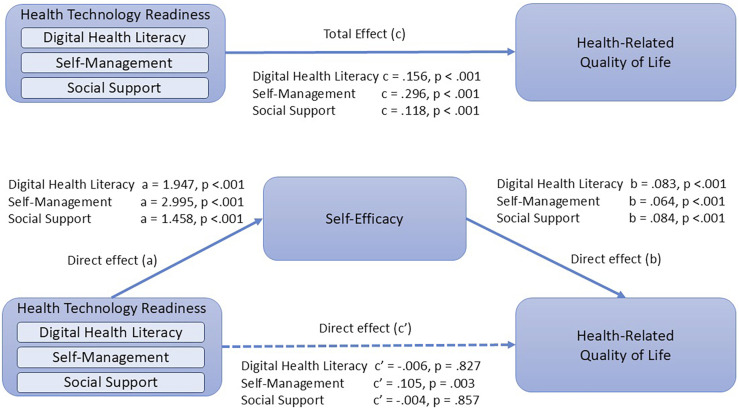
Relationships between the domains of health technology readiness, self-efficacy, and health-related quality of life.

The results of the mediation analysis with PROCESS macro are presented in Table 5 and Figure 2. The total effect of DHL on HR-QoL was statistically significant (*B* = .156, 95% CI: [.095, .218]). However, after including the mediator, self-efficacy, in the model, the direct association was statistically non-significant (*B* = -.006, 95% CI: [-.062, .050]), but the results of a significant indirect association between DHL and HR-QoL (*B* = .163, 95% CI: [.114, .218]), are consistent with mediation with self-efficacy as the mediating variable. Together, DHL and self-efficacy explained 41.5% of the variance of HR-QoL.

**Table 5.** Mediation analysis of self-efficacy in the association of health technology readiness and health-related quality of life-QoL.

​	Self-efficacy in the association of DHL^e^ and HR-QoL^f^			Self-efficacy in the association of self-management and HR-QoL^f^			Self-efficacy in the association of social support and HR-QoL^f^		
​	Total effect (C)	Direct effect (C')	Indirect effect (a*b)	Total effect (C)	Direct effect (C')	Indirect effect (a*b)	Total effect (C)	Direct effect (C')	Indirect effect (a*b)
***B*** ^a^	.156	-.006	.163	.296	.105	.191	.118	-.004	.122
**SE** ^b^	.031	.028	.021	.028	.036	.035	.023	.021	.020
**95% CI** ^c^	.095- .218	-.062 - .050	.114 - .218	.242 - .351	.035 - .176	.128 - .266	.073 - .164	-.046 - .038	.086 - .163
***P* value** ^d^	<.001	.827	<.001	<.001	.003	<.001	<.001	.857	<.001
percent mediated (a*b/c)	104,5 %			64.5 %			103.4 %		

^a^Unstandardized coefficient.

^b^Standard error.

^c^Confidence Interval.

^d^*P*<.05 is considered statistically significant.

^e^Digital health literacy.

^f^Health related quality of life.

Similarly, the total effect of social support on HR-QoL was statistically significant (*B* = .118, 95% CI: [.073, .164]), but the direct association was insignificant (*B =* -.004, CI 95%: [-.046, .038]). The indirect association was statistically significant (*B* = .122, CI 95%: [.086, .163]) after including self-efficacy as a mediating variable in the model. Together, social support and self-efficacy explained 40.3% of the variance in HR-QoL. In contrast, concerning self-management, the direct association with HR-QoL (*B=*.105, 95% CI: [.035, .176]), and the indirect association through self-efficacy (*B=*.191, 95% CI: [.128, .266], *P*>.001) were statistically significant, consistent with partial mediation.

## Discussion

### Principal results and comparison to prior research

This study aimed to investigate the relationship between health technology readiness and HR-QoL and the role of self-efficacy as a possible mediator between health technology readiness and HR-QoL among preference-sensitive neurological conditions. Our research was based on the READHY instrument, which aims to give a holistic view of individuals’ health technology readiness, including DHL, self-management, and social support^37^. To our knowledge, no prior research has examined the relationship between health technology readiness and HR-QoL. Although health technology readiness provides a comprehensive understanding of users’ ability to use health technology, it also describes the degree to which this ability is enabled through its aspects^37^. As the READHY instrument provides information on health technology readiness as a summary of the variables included in each aspect, as described in the methods, our results are reported separately for each aspect. Our results show that all aspects of health technology readiness had statistically significant relationships with HR-QoL, however, only self-management and HR-QoL had a statistically significant direct association. DHL and social support had only indirect significant associations with HR-QoL, consistent with mediation by self-efficacy. This indicates that the relationships between the aspects of health technology readiness and HR-QoL are complex.

Our findings of a statistically significant relationship between DHL and HR-QoL align with earlier research in an Iranian community,^25^ among older adults,^24,28,30,31^ and individuals who have undergone kidney stone treatment^26^ or prostatectomy.^27^ However, the mediation analysis indicates that this association may be mediated by self-efficacy, which is consistent with Xie et al.’s^30^ conclusion that self-efficacy is a possible mediator between DHL and health outcomes such as HR-QoL. In our research, the direct association between DHL and HR-QoL was negative, although not significant, indicating that higher DHL is associated with lower HR-QoL. Similarly, Silverstein et al.^21^ reported that higher DHL was associated with worse asthma-related quality of life. They suggest it may be related to the active use of DHS to seek more information about their condition.^21^ Furthermore, the significant positive indirect association between DHL and HR-QoL, with self-efficacy as a mediating variable, is consistent with mediation, indicating that the self-efficacy may play a role in the positive association between DHL and HR-QoL.

Similar to the relationship between DHL and HR-QoL, our results indicate a significant positive relationship between social support and HR-QoL, confirming the previous research on the positive relationship between social support and HR-QoL among people with MS^46,68^ and, more generally, social support as a social determinant positively associated with HR-QoL.^44^ Furthermore, the direct relationship between social support and HR-QoL is negative, although insignificant, whereas the indirect relationship via self-efficacy is consistent with mediation. The positive relationship between social support and self-efficacy has also been recognized in patients with a kidney transplant^69^ and multiple chronic diseases.^45^ This aligns with Bandura’s^34^ statement that social support may be effective in promoting the successful adoption of healthy habits only when it increases one’s self-efficacy.

Our results show a significant, positive relationship between self-management and HR-QoL. Mediation analysis showed that this relationship is both direct and indirect, with self-efficacy as the mediating variable in this association. As these relationships are both significant, the results are consistent with partial mediation. Although we could not locate research using the same instrument, Pozza et al.^70^ reported that, among self-management skills, only lower emotional distress was associated with higher physical and mental quality of life, and constructive attitudes and approaches were positively associated with physical quality of life. Both of these self-management skills were included in our instrument. This finding of a mediating role for self-efficacy is supported by reviews reporting a significant positive relationship between self-management and self-efficacy, despite a positive, albeit nonsignificant, relationship between digital self-management interventions and HR-QoL.^47,48^

Our overall results indicate that all three aspects of health technology readiness are positively related to HR-QoL; however, these relationships are consistent with mediation, with self-efficacy as the mediating variable. Research on the separate aspects of health technology readiness supports our findings, as each aspect has been reported to have a positive association with HR-QoL and a statistically significant positive relationship with self-efficacy. Furthermore, self-efficacy has been reported as one of the patient-centered predictive factors of HR-QoL among people with type 2 diabetes^71^ and as a mediator between DHL and HR-QoL,^30^ supporting our findings of self-efficacy’s role as a mediator. This aligns with the conclusions of Silverstein et al.^21^ and Stellefson et al.,^29^ who found that adequate DHL is a crucial factor in increasing patient engagement with digital health applications that support self-management of chronic diseases. Especially when aiming to promote HR-QoL.^29^ In addition to adequate health literacy, DHL is crucial for empowering individuals with preference-sensitive conditions to participate in decision-making and self-care.^26,27^ This suggests that when planning interventions to promote DHL in individuals with chronic conditions, it would be beneficial to combine these interventions with support for self-motivation and self-management of their condition as suggested by Bandura.^34^ Furthermore, HR-QoL is often used as an outcome measure of digital interventions,^35^ but DHL, as the ability to use health-related information in digital form, does not always directly result in the use of DHS. Lived experience of disease burden may explain diversity in the adaptation of digital self-management tools.^72^ These results suggest that, in addition to having the knowledge to use health information in digital form, there is a need for self-efficacy, one’s confidence in the ability to manage health-related habits, to fully benefit from DHL in the promotion of HR-QoL.

### Limitations

We recognize that this study has several limitations. First, due to the cross-sectional study design, causality between health technology readiness, self-efficacy, and HR-QoL cannot be determined. Longitudinal research is necessary to gain a deeper understanding of potential causality. Furthermore, subjective self-reports were used to measure key variables. Self-reports describe individuals’ subjective perceptions rather than their objective clinical status. This may introduce variability and person-specific interpretation. Future research should combine subjective measures with physiological indicators to yield a comprehensive dataset and a better understanding of individual differences.

Third, it is essential to note the limited size of the study population when generalizing the results. Although the questionnaire was distributed through patient organizations and a neurology outpatient clinic, the sample size remained limited. Furthermore, in this study, sample size calculation was not feasible due to the recruitment strategy. The survey invitation was disseminated through patient organizations via social media and newsletters. Therefore, the total number of individuals with epilepsy or MS who had access to the invitation could not be determined. Consequently, it was not possible to conduct an a priori sample size calculation. Instead, we aimed to reach as many eligible participants as possible through these channels. This may affect the generalization of the results.

Fifth, a pilot of the entire questionnaire was not conducted, as it was built from validated, widely used instruments. However, this may have limited the ability to identify issues related to the integration and flow of the combined questionnaire.

In addition, voluntary study design may have introduced selection bias, with an overrepresentation of individuals interested in the use of health technology. However, the proportion of users of DHS among the respondents is similar to another Finnish research on people with MS^17^ and in Finnish population general.^73^ The questionnaires were also distributed in paper format at the neurology outpatient clinic to better reach those less familiar with technology. In addition, the self-selection bias in the gender distribution should be noted when generalizing the results, as 78.9% of respondents were female, which is consistent with the distribution of people with MS but inconsistent with the typical gender distribution of people with epilepsy.

### Implications for practice and future research

Based on our results, the future development of DHS and digital health interventions should include elements to enhance self-efficacy and self-management. This could include e.g. personalization and the option to report patient-generated health-data (e.g. symptoms diary).

Due to the cross-sectional design, these results provide a preliminary understanding of the relationships among the three aspects of health technology readiness, self-efficacy, and HR-QoL, and longitudinal research is needed to examine possible causal relationships. In addition, the study population was limited, and further research is required to test the findings and their implications.

In addition, due to the limited number of respondents, the data were used to explore relationships among health technology readiness, self-efficacy, and HR-QoL at a general level. Further research with disease-specific measures is required to further investigate the findings.

## Conclusions

The key finding was that all three aspects of health technology readiness were significantly associated with HR-QoL; however, these relationships were consistent with mediation, with self-efficacy as the mediating variable. This suggests that the mechanisms underlying DHL’s, social support’s, and self-management’s promotional effect on HR-QoL may be mediated by the motivational impact of self-efficacy. Therefore, the roles of self-efficacy and all three aspects of health technology readiness should be considered when planning digital health interventions to fully achieve the benefits of digital health.

The results of this study also highlight the advantages of using a comprehensive measure of health technology readiness, such as the READHY instrument, to gain a holistic view of aspects related to digital health use. This further supports the planning of digital health interventions. However, more research is needed to further investigate these relationships. Research is also needed to examine whether including an assessment of self-efficacy could support the information acquired from the self-management of health technology readiness.

## Data Availability

The datasets generated and/or analyzed during the current study are not publicly available due to the project’s data management plan but are available from the corresponding author upon reasonable request.*
